# Unstable elbow dislocations: the description and cadaveric feasibility study of a new surgical technique

**DOI:** 10.1051/sicotj/2015023

**Published:** 2015-08-05

**Authors:** Mark Harris, Timothy Bishop, Jason Bernard

**Affiliations:** 1 Department of Orthopaedic surgery, St Georges NHS Foundation Trust Blackshaw Road Tooting, London SW17 0QT UK

**Keywords:** Elbow dislocation, Surgical stabilisation

## Abstract

*Introduction*: A small proportion of simple elbow dislocations are grossly unstable and joint congruence is not maintained after reduction. In this rare situation operative treatment is indicated. We describe a new intra articular reconstruction that utilises a slip of triceps tendon to provide immediate stability to the elbow.

*Methods*: We assessed 20 cadaveric elbows, measuring the length of triceps tendon available and required to complete the reconstruction. We then sequentially sectioned the ligamentous stabilisers of an elbow before performing the new technique. We measured the displacement and angulation possible at the elbow before and after the reconstruction.

*Results*: All 20 elbows had sufficient triceps tendon length to complete the new technique. Prior to the reconstruction greater than 30 mm of joint distraction and 90 degrees varus or valgus angulation was possible. Following the reconstruction it was not possible to re-dislocate the elbow. Only 2 mm of joint distraction and 10 degrees of varus or valgus angulation were possible with the triceps graft fixed in position.

*Discussion*: This novel technique elegantly avoids many of the problems associated with current methods. We have demonstrated that it is technically feasible and easy to perform with minimal equipment requirements or costs.

## Introduction

The elbow is the second most frequently dislocated large joint. The dislocation is classified as simple or complex depending on the presence or absence of an associated fracture [1]. Simple dislocations have an incidence of 5–6 per 100,000 [2, 3]. The vast majority of simple dislocations can be reduced closed with sedation and will remain reduced and stable [4]. Early active range of motion within 1–2 weeks has been shown to be safe and produce good outcomes compared with prolonged immobilisation [5]. A small proportion of simple dislocations are grossly unstable and do not remain reduced with standard non-operative treatment. In this scenario several operative techniques have been described including open collateral ligament repair or reconstruction [6], fixed or hinged external fixators [7] and trans-articular pinning [8, 9].

We propose a new surgical technique and assess its feasibility in a cadaveric study.

## Materials and methods

### The technique

This technique utilises the harvest of a central strip of triceps tendon which is distally based and remains attached at its insertion. The central triceps strip is passed through a fenestration made in the olecranon fossa and fixed to the coronoid process to construct a complete osseo-tendonous ring (coronoid, olecranon and triceps tendon) that holds the ulna congruent with the trochlea of the distal humerus. Thus the joint is stabilised and a normal relationship between the ulna and the elbow axis of rotation is maintained throughout a full range of motion.

**Figure 1.**
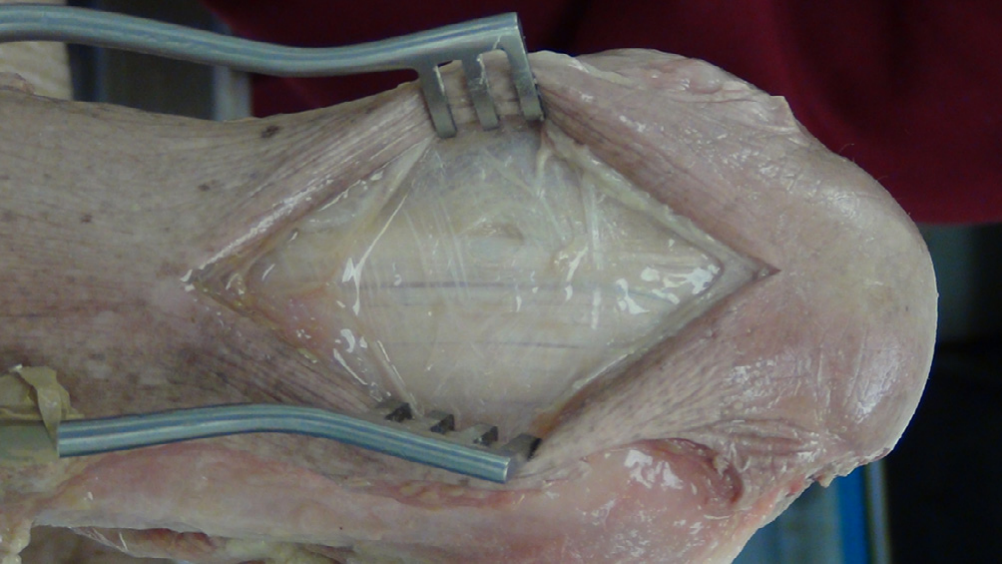
Exposure of triceps tendon.

**Figure 2.**
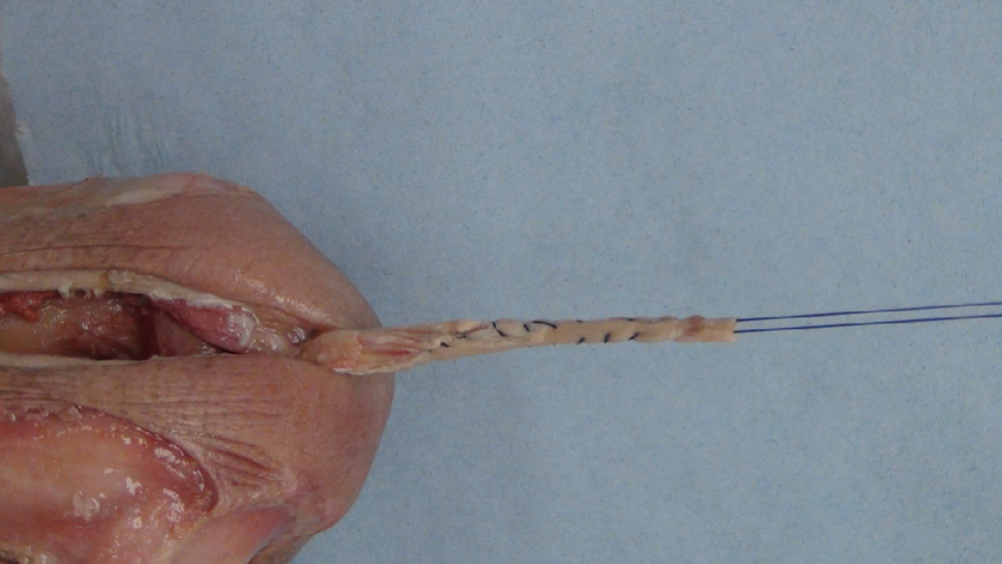
Central strip of triceps tendon harvested and whip stitched.

**Figure 3.**
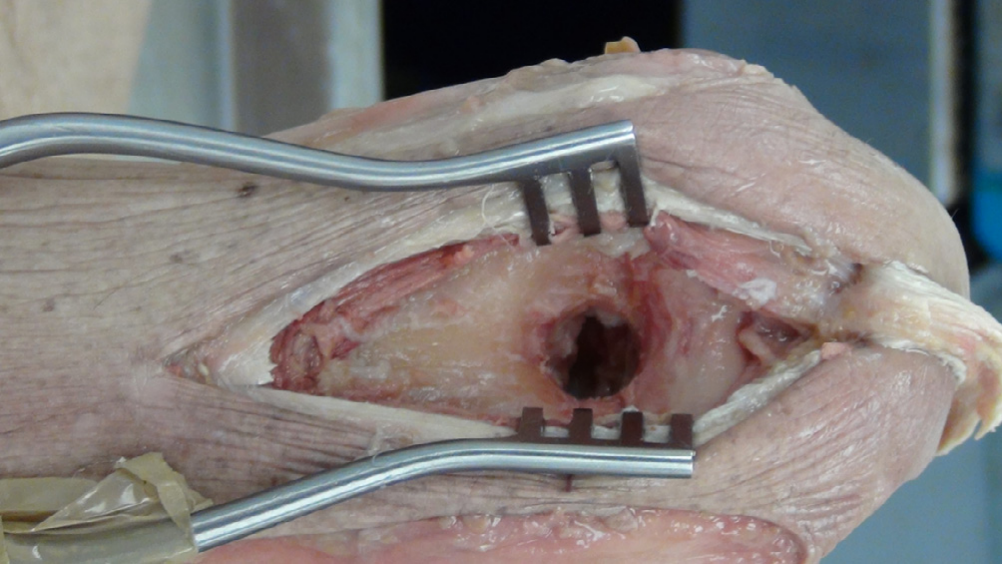
Fenestration of the olecranon fossa.

**Figure 4.**
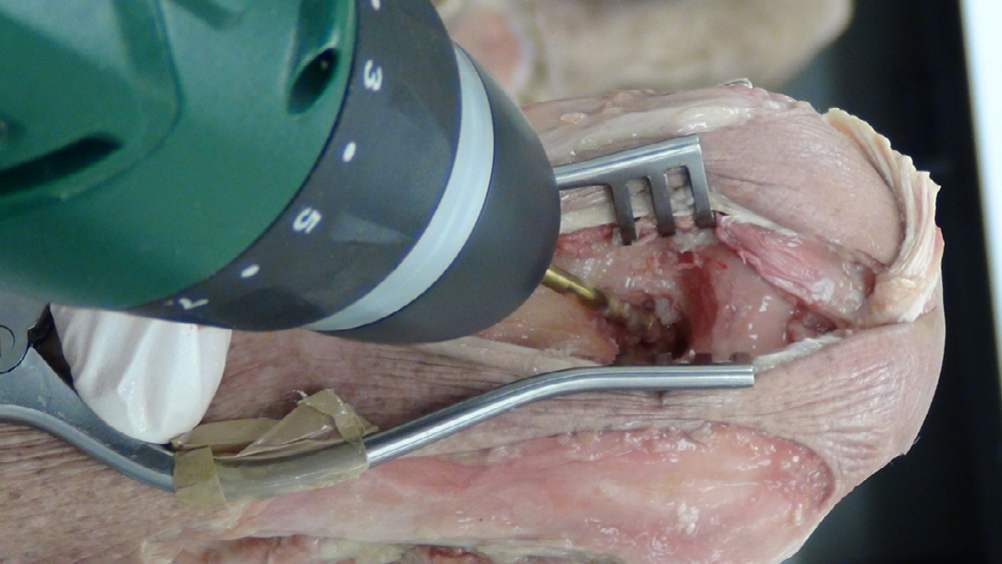
Drilling of the graft tunnel in the ulna through the olecranon fossa.

**Figure 5.**
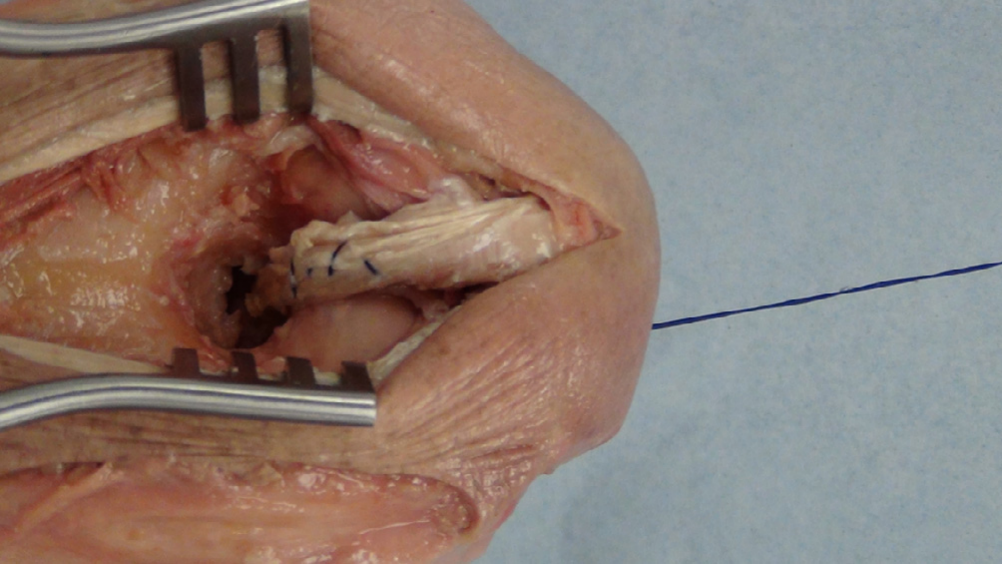
Posterior view of the graft in its final position.

**Figure 6.**
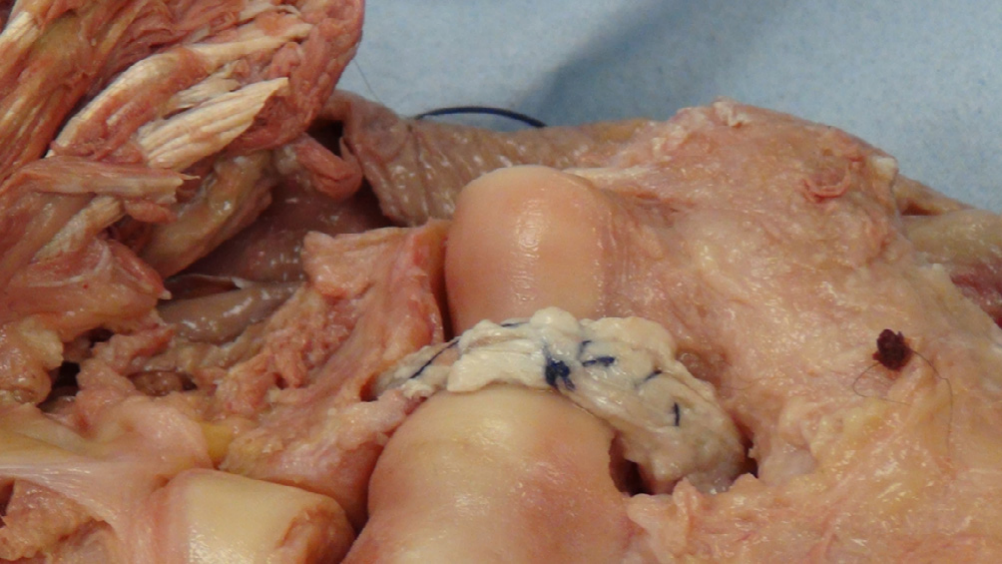
Anterior view of the graft in its final position.

A posterior longitudinal incision is made from the tip of the olecranon and extended 10 cm proximally. The triceps tendon is exposed and a 4 mm wide central strip extending proximally from olecranon to the musculotendonous junction is marked out (Figure 1). The tendon strip is divided proximally at the musculotendonous junction and longitudinally to its insertion. The insertion is left intact. The slip of harvested tendon is then whip stitched with the tails of the suture left long at the free end of the tendon (Figure 2). The posterior aspect of the humerus is then visible through the triceps split. The posterior olecranon fossa is cleared and a fenestration is made with a 4 mm drill to access the anterior joint space (Figure 3). Access to the anterior aspect of the joint through a window in the olecranon fossa is described in the Outerbridge-Kashiwagi (OK) method for the debridement of osteophytes in degenerative joint disease [10]. The elbow is flexed so that the tip of the coronoid process is visible through the fossa. A 4 mm tunnel is then drilled beginning at the tip of the coronoid process and traversing the ulna to exit through the dorsal cortex of the ulna (Figure 4). A small incision is made over the tip of the drill at the point where it penetrates the dorsal cortex of the ulna. The free ends of the whip stitch are passed through the fenestration in the olecranon fossa and through the tunnel in the ulna with the aid of a suture passer. The tendon is pulled through the tunnel, tensioned and fixed by tying the whip stitch through a transverse 2.5 mm drill hole in the ulna. This completes the osseo-tendonous ring (coronoid, olecranon and triceps tendon) giving immediate stability (Figures 5 and 6). The isometric relationship between the articular proximal ulna and the axis of rotation of the elbow is restored allowing a full range of movement. The longitudinal split in the triceps tendon is then closed with sutures.

### Cadaveric study

**Figure 7.**
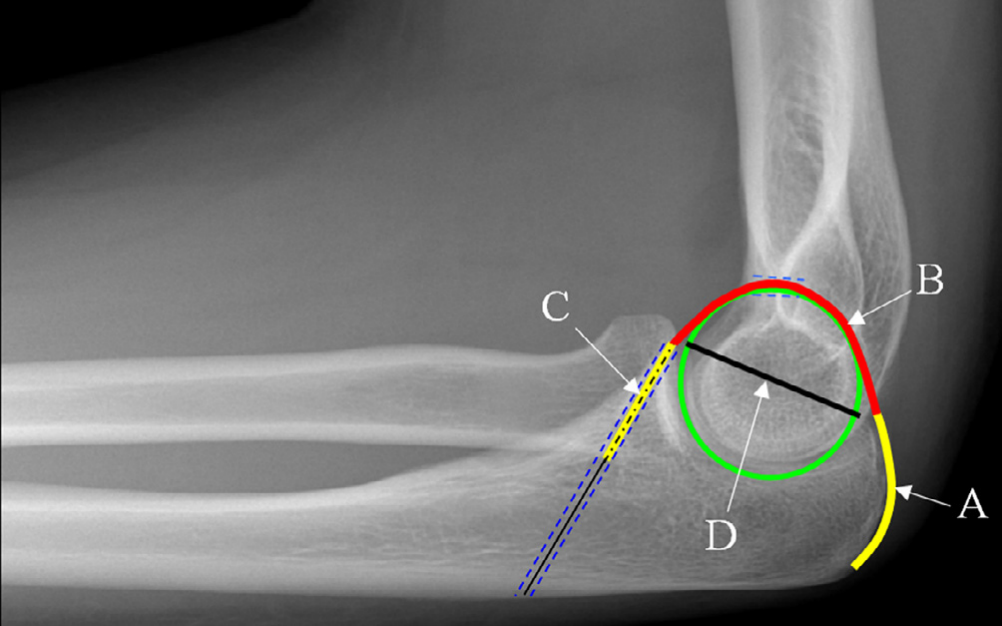
Lateral radiograph of an elbow with superimposed schematic of the reconstruction. The length of tendon required = A + B (1/2πD) + C.

The feasibility of our new technique requires sufficient triceps tendon length to complete the osseo-tendonous ring. The length required is the sum of three sections (Figure 7). The first part (A) is the insertion on the olecranon. The second (B) is the intra-articular portion which passes from the olecranon through the olecranon fossa and to the coronoid. The third part is in the bone tunnel in the coronoid process and is fixed at 15 mm. We chose 15 mm as the length of graft in the tunnel because this has been reported as a safe length in anterior cruciate ligament reconstruction [11, 12]. We dissected 10 cadavers (20 elbows). The distance A was measured with callipers. The distance B was calculated on the basis of an observation that a line drawn from the tip of the coronoid to the tip of the olecranon (D) passes through the axis of rotation of the elbow. This represents the diameter of our proposed osseo-tendonous ring. The length of B is assumed to be half the circumference of the ring (1/2πD). The distance between the tip of the coronoid and the triceps insertion on the olecranon was measured with callipers.

The technique was then performed on a single cadaveric elbow in which all ligamentous stabilisers were sequentially sectioned to mimic the clinical scenario of a grossly unstable elbow dislocation. Joint distraction and stressed varus and valgus angulation were recorded before and after the reconstruction with a rule and a goniometer.

## Results

All 20 cadaveric elbows were assessed. In all the elbows there was sufficient triceps tendon length to perform the technique. The mean length of triceps tendon available for use was 106 mm. The mean length of the tendon insertion A was 22 mm. The mean distance (D) from coronoid tip to triceps insertion on the olecranon was 34 mm. This gives a calculated tendon length (1/2πD) of 53 mm for the intra-articular portion (B) of the graft. The mean total length of graft required (A + B + C) was 91 mm (Table 1).

**Table 1.** Lengths of triceps tendon required and available in 20 cadaveric elbows..

	Lengths (mm) of tendon required and available for reconstruction				
ELBOW	A	D	B (1/2πD)	Length required (A + B + 15 mm)	Length available
1	23	33	52	90	134
2	24	35	54	93	99
3	19	36	56	90	98
4	21	33	52	87	110
5	20	33	52	88	121
6	25	34	54	94	95
7	25	35	55	95	102
8	24	35	55	93	102
9	24	36	56	95	104
10	21	31	49	85	90
11	21	34	54	90	126
12	19	32	50	84	95
13	23	34	54	91	93
14	25	35	55	95	115
15	21	34	54	90	115
16	20	30	46	81	98
17	26	36	57	98	105
18	23	34	53	90	118
19	23	34	53	90	99
20	24	33	52	91	93
Mean all (*SD*)	22	34	53	91 (4)	106 (12)

Prior to the reconstruction gross elbow instability was demonstrated. More than 30 mm of joint distraction was possible and greater than 90 degrees varus or valgus angulation was possible. Following the reconstruction it was not possible to re-dislocate or sublux the ulnohumeral joint regardless of the elbow position from full extension to full flexion. Only 2 mm of joint distraction was and 10 degrees of varus or valgus angulation were possible with the triceps graft fixed in position. The graft tracked nicely in the trochlea grove with no impingement.

## Discussion

A simple elbow dislocation that spontaneously re-dislocates following closed reduction and appropriate stabilising manoeuvres (elbow flexion and forearm pronation) is a rare problem. Failure to restore joint congruence is likely to be associated with stiffness, reduced range of motion, instability and future degenerate change [13]. Operative treatment is therefore indicated. There are various operative techniques described in the literature but there is a lack of published evidence to support any one particular treatment method. Broadly speaking these can be divided into techniques which maintain the reduction by static or dynamic means. Static stabilisation of the elbow is relatively technically simple and has been described with the use of external fixation or trans-articular pinning. The primary disadvantage of this static approach is in the tendency of the elbow to stiffen following severe injury. This is exacerbated by prolonged immobilisation following dislocation and is associated with poorer outcomes than early functional rehabilitation [14–17]. There is an argument for static reduction particularly trans-articular pinning in patients who are not fit for more prolonged or technically difficult surgery or are being treated in “resource poor” environments [18]. Dynamic stabilisation aims to avoid the problems of immobilisation by allowing early functional rehabilitation. Good results have been reported [19, 20]. Most commonly, dynamic stabilisation is achieved with soft tissue repair or reconstruction or hinged external fixation. It has been shown that in unstable simple elbow dislocation most if not all the primary soft tissue stabilisers of the joint are ruptured [21]. The pathomechanics of dislocation proposed by Horii describes sequential failure of the soft tissues from lateral to medial. Stage 1 begins with the lateral collateral ligament complex (LCLC) which is comprised of the lateral ulnar collateral ligament (LUCL), the radial collateral ligament (RCL) and the annular ligament. Stage 2 is the anterior capsular structures. Stage 3 is rupture of the medial collateral ligament and is divided into three. In 3A the posterior bundle of the medial ulnar collateral ligament (MUCL) is ruptured but the anterior bundle is intact. In 3B the anterior bundle is ruptured and in 3C the elbow remains unstable after reduction even in 90 degrees of flexion [22]. Additionally the common flexor and extensor origins are frequently avulsed from the medial and lateral epicondyles. Deciding which of these structures to repair adds complexity to the management. The importance of the anterior band of the MUCL and the LUCL has been highlighted by O’Driscoll [23]. However, there is no single protocol to guide the surgeon on which structures should be repaired and in what order. Additionally the repair or reconstruction of the collaterals must be anatomical to allow unrestricted elbow flexion and extension around its axis of rotation. Malpositioning of the isometric LCL or the non-isometric MUCL [24] will result in stiffness or instability depending on the position of the elbow during tensioning of the repair. Furthermore, the severity of injury to the ligaments being repaired may necessitate augmentation with free tendon graft or skeletal support with hinged external fixators. Hinged external fixators must be applied exactly aligned with the axis of rotation of the joint and are associated with high rates of complications, particularly infection [25].

We believe that our novel technique elegantly avoids many of the problems associated with current methods. We have demonstrated that it is technically feasible and easy to perform with minimal equipment requirements or costs. There is no need for metal insertion or its subsequent removal. The osseo-tendonous ring constructed around the trochlea provides immediate stability with concentric reduction such that the difficulties of aligning the ulna with the axis of rotation of the elbow are negated. The triceps tendon is only exceptionally rarely injured in simple elbow dislocation [26] and so the graft is strong and should avoid the need for additional stabilisation of the joint. In addition, the graft remains attached to its insertion on the olecranon and remains vascularised.

This is primarily a feasibility study to demonstrate that our idea is technically possible. We think that this is a responsible approach to take before introducing a new procedure to patients. It does not prove clinical applicability of the technique or superiority to current conventional methods. There are potential drawbacks specific to this technique. The triceps tendon may be at risk of donor site morbidity including pain and weakness similar to that seen following patella tendon harvest for ACL reconstruction [27]. The OK method has a reported risk of distal humerus fracture [28] and heterotopic ossification [29]. Although feasible on all unstable simple dislocations, further biomechanical testing including cyclic loading of the tendon would be required before the potential applicability of this technique is known. Initially we believe that the most appropriate role for this procedure is on the cohort of patients who would currently be considered unsuitable for dynamic ligamentous repair or external fixation. This cohort may be medically unsuitable for prolonged surgery or assessed as not able to tolerate external fixation. Their healing potential may be reduced due to comorbidities such as diabetes or medications such as steroids. We would also advocate this technique in the “resource poor” environment.

These patients are typically offered trans-articular pinning or non-operative treatment and it is in this setting that we believe our new technique will offer them most advantage.

## Conflict of interest

MH, TB and JB declare no conflict of interest in relation with this paper.

## Online material

**Video 1** – Post reconstruction stability. **Video 2** – Pre reconstruction instability.
